# A declaration on the value of experiential measures of food and water insecurity to improve science and policies in Latin America and the Caribbean

**DOI:** 10.1186/s12939-023-01956-w

**Published:** 2023-09-05

**Authors:** Hugo Melgar-Quiñonez, Pablo Gaitán-Rossi, Rafael Pérez-Escamilla, Teresa Shamah-Levy, Graciela Teruel-Belismelis, Sera L. Young, the Water Insecurity Experiences-Latin America, the Caribbean (WISE-LAC) Network, Monica Ancira-Moreno, Antonio Barbosa-Gomes, Hilary Bethancourt, Mauro Brero, Soraya Burrola, Alejandra Cantoral, Haydee Cárdenas-Quintana, Julio Casas-Toledo, Sara Eloisa Del Castillo, Marti Del Monte-Vega, Mauro Del Grossi, Claire Dooley, Olga Espinal-Gomez, Gabriela Fajardo, Adriana Flores-Díaz, Edward A. Frongillo, Olga García, Erika Garcia-Alberto, María Girona, Daniela Godoy-Gabler, Mauricio Hernández-Fernández, Gonzalo Hernandez-Licona, Sonia Hernandez-Cordero, Alan Hernandez-Solano, Martha Patricia Herrera-González, Vania Lara-Mejia, Gerardo Leyva-Parra, Charlotte MacAlister, Édgar Martínez-Mendoza, Carla Mejia, Joshua Miller, Rebeca Monroy-Torres, Verónica Mundo-Rosas, Alicia Muñoz-Espinosa, Sara Nava-Garcia y Rodriguez, Lynnette Neufeld, Juan Nuñez, Poliana Palmeira- de Araújo, Israel Rios-Castillo, Alberto Rodríguez-Abad, Rosana Salles-Costa, Daniela Serrano-Campos, Isidro Soloaga, Brenda Tapia-Hernandez, Jefferson Valencia, Mireya Vilar-Compte, Paloma Villagómez-Ornelas

**Affiliations:** 1 School of Human Nutrition, McGill University, Montreal, QC Canada; 2 Instituto de Investigaciones Para El Desarrollo Con Equidad, Universidad Iberoamericana, Prolongación, Av. P.º de La Reforma 880, Santa Fe, Álvaro Obregón, Ciudad de México, 01219 México; 3 Department of Social and Behavioral Sciences, Yale School of Public Health, Yale University, New Haven, CT USA; 4 Centro de Investigación en Evaluación Y Encuestas, Instituto Nacional de Salud Pública, Cuernavaca, Morelos México; 5 Department of Anthropology & Institute for Policy Research, Northwestern University, Evanston, IL USA

**Keywords:** Food insecurity, Water insecurity, Food security, Water security, Sustainable development goals, Latin American & Caribbean, Measurement, Indicator, Scales

## Abstract

**Background:**

Water security is necessary for good health, nutrition, and wellbeing, but experiences with water have not typically been measured. Given that measurement of experiences with food access, use, acceptability, and reliability (stability) has greatly expanded our ability to promote food security, there is an urgent need to similarly improve the measurement of water security. The Water InSecurity Experiences (WISE) Scales show promise in doing so because they capture user-side experiences with water in a more holistic and precise way than traditional supply- side indicators. Early use of the WISE Scales in Latin America & the Caribbean (LAC) has revealed great promise, although representative data are lacking for most of the region. Concurrent measurement of experiential food and water insecurity has the potential to inform the development of better-targeted interventions that can advance human and planetary health.

**Main text:**

On April 20–21, 2023, policymakers, community organizers, and researchers convened at Universidad Iberoamericana in Mexico City to discuss lessons learned from using experiential measures of food and water insecurity in LAC. At the meeting’s close, organizers read a Declaration that incorporated key meeting messages. The Declaration recognizes the magnitude and severity of the water crisis in the region as well as globally. It acknowledges that traditional measurement tools do not capture many salient water access, use, and reliability challenges. It recognizes that the WISE Scales have the potential to assess the magnitude of water insecurity more comprehensively and accurately at community, state, and national levels, as well as its (inequitable) relationship with poverty, poor health. As such, WISE data can play an important role in ensuring more accountability and strengthening water systems governance through improved public policies and programs. Declaration signatories express their willingness to promote the widespread use of the WISE Scales to understand the prevalence of water insecurity, guide investment decisions, measure the impacts of interventions and natural shocks, and improve public health.

**Conclusions:**

Fifty-three attendees endorsed the Declaration – available in English, Spanish and Portuguese— as an important step to making progress towards Sustainable Development Goal 6, “Clean Water and Sanitation for All”, and towards the realization of the human right to water.

**Supplementary Information:**

The online version contains supplementary material available at 10.1186/s12939-023-01956-w.

## Introduction

On April 20–21, 2023 a scientific meeting was convened in Mexico City to discuss lessons learned from using experiential measures of food and water insecurity in the Latin American and Caribbean (LAC) region [1–3]. It brought together scientists, government officials, and other key actors from Brazil, Canada, Chile, Colombia, Guatemala, Honduras, Mexico, Italy, Panama, Peru, Uruguay, the United Kingdom, and the United States. The meeting was organized by scholars from Universidad Iberoamericana, the Mexican National Institute of Public Health (INSP), Northwestern University, McGill University, and Yale University; and comprised scholars from the Autonomous University of Querétaro, the Universidad de la República de Uruguay, University of North Carolina-Chapel Hill, and the London School of Hygiene and Tropical Medicine. Other attendees represented United Nations organizations like the Food and Agriculture Organization, UNICEF, United Nations University Institute for Water Environment and Health, and the World Food Program. Participants from the Mexican government included the Ministry of Equality and Inclusion from the Mexican state of Nuevo León and the Institute for Democratic Prospective Planning of Mexico City. Likewise, research and evaluation entities were represented by the National Institute of Statistics and Geography (INEGI), the National Evaluation Council (CONEVAL) and the INSP. Non-governmental organizations in attendance were Action Against Hunger, Innovations for Poverty Action, Brazil’s Articulação no Semiárido Brasileiro, Colombia´s Alianza Universitaria por el Derecho Humano a la Alimentación Adecuada, and the Observatorio del Derecho a la Alimentación de América Latina y el Caribe; other international development entities were the Inter-American Development Bank, the US Agency for International Development, and the US Embassy in Mexico.

The primary aim of the meeting was to appraise the value that experiential measures bring to the key development issues of food insecurity and water insecurity, which broadly refer to the unreliable availability or access to food and water. These measures, which capture user-side experiences with access, use, quality, and reliability of key resources, provide more nuanced insights than observational measures. For example, food security was once tracked using only supply-side indicators, such as food balance sheets [4, 5]. The evolution of food security indicators to include a measure of experiences with the reliability of food access and use, i.e., “user-side indicators” has been invaluable for understanding disparities in food security, i.e., when food might be available in the community but not accessible to a family or an individual [6]. Indeed, experiential measures of food security are sufficiently valued that they are now an indicator for Sustainable Development Goal 2 (“Zero Hunger”) [7]. Lessons learned from the global implementation of the Water InSecurity Experiences (WISE) Scales in the last several years suggests that experiential measures of water insecurity similarly have added value because they reveal issues than more traditional “supply-side” indicators, like m^3^ of water per capita and water infrastructure, could not [8–10].

**Fig. 1:**
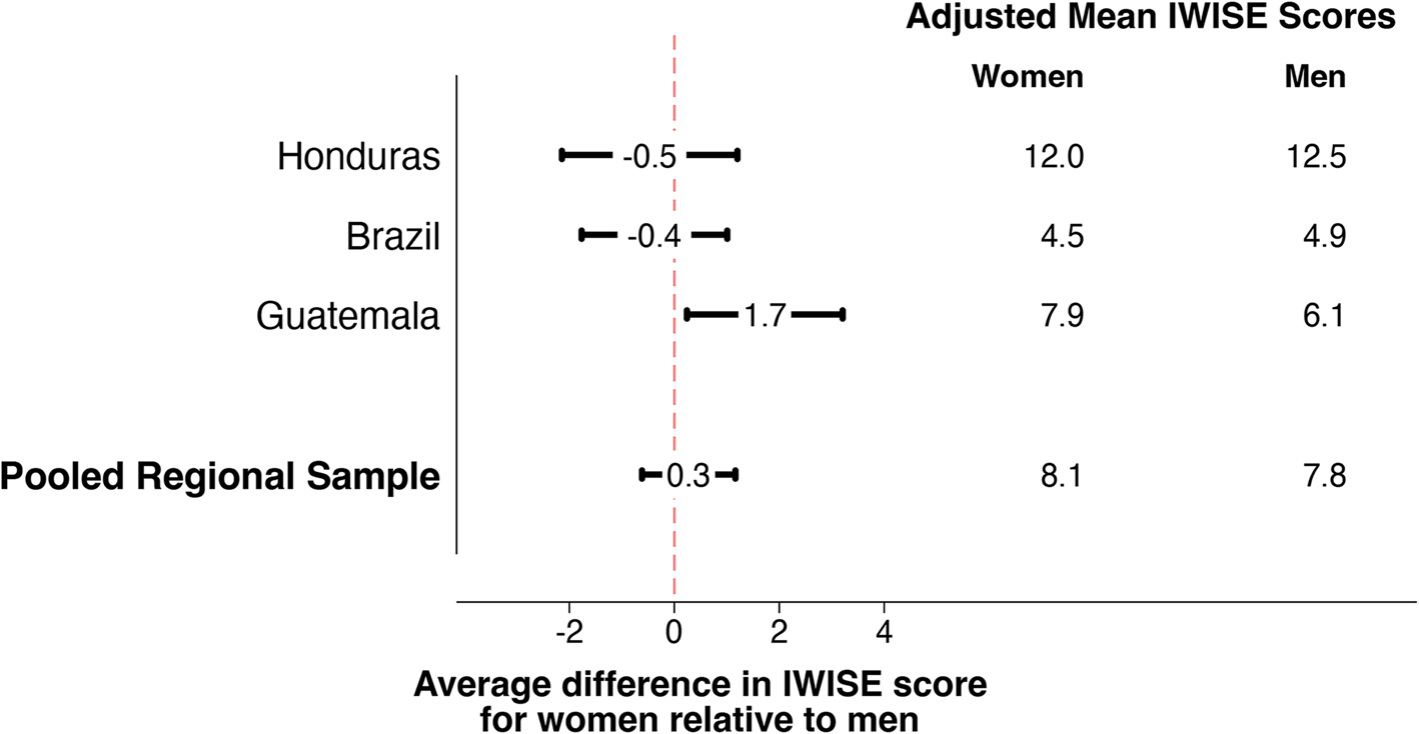
Differences in IWISE scale scores between women and men, adjusted for key sociodemographic variables, using nationally representative samples of individuals from the 2020 Gallup World Poll (*n* = 2,972; Honduras *n* = 949; Brazil *n* = 974; Guatemala *n* = 1049). Datapoints indicate β coefficients and horizontal lines indicate 95% CIs. Coefficients and 95% CIs for each country were estimated from multivariable linear regression models that regressed IWISE score on an indicator of female gender (reference is male gender), controlling for urbanicity, income bracket, difficulty getting by on household income, employment status, age, household size, marital status, and education (further adjusting for country in the pooled regional model). The adjusted mean IWISE scores for women and men are the marginal means obtained from these models when all other variables are held constant at their means. Due to rounding, the differences in marginal means can differ from the coefficients by 1 decimal point. IWISE = Individual Water Insecurity Experiences. This figure is replicated, with permission, from [13]

Two key advantages of experiential measures of water insecurity over the more traditional, supply-side indicators are that they are more holistic and more precise. The WISE scales are more holistic because they capture experiences with water beyond sufficiency for drinking, such as hygiene and cooking, as well as key psychosocial dimensions like worry and anger. They are more precise because they can be applied at the household level (Household Water Insecurity Experiences (HWISE) Scale [11]) and at the individual level (Individual Water Insecurity Experiences (IWISE) Scale [12]). As such, the data they generate allow for the exploration of differences by important household characteristics like urbanicity and wealth, and individual characteristics like gender, age, and ethnicity. For example, in a nationally representative study of water insecurity experiences among adult individuals in low and middle-income countries, multivariate models revealed that gender was a very strong covariate of water insecurity in some countries [13]. Here we replicate the differences in water insecurity by gender for the LAC countries in that study (Fig. 1). Interestingly, water insecurity was not meaningfully different between women and men in neither Honduras nor Brazil, but was so in Guatemala, where women scored approximately 1.7 points higher on the IWISE Scale (range 0–36), indicating greater water insecurity.

**Fig. 2:**
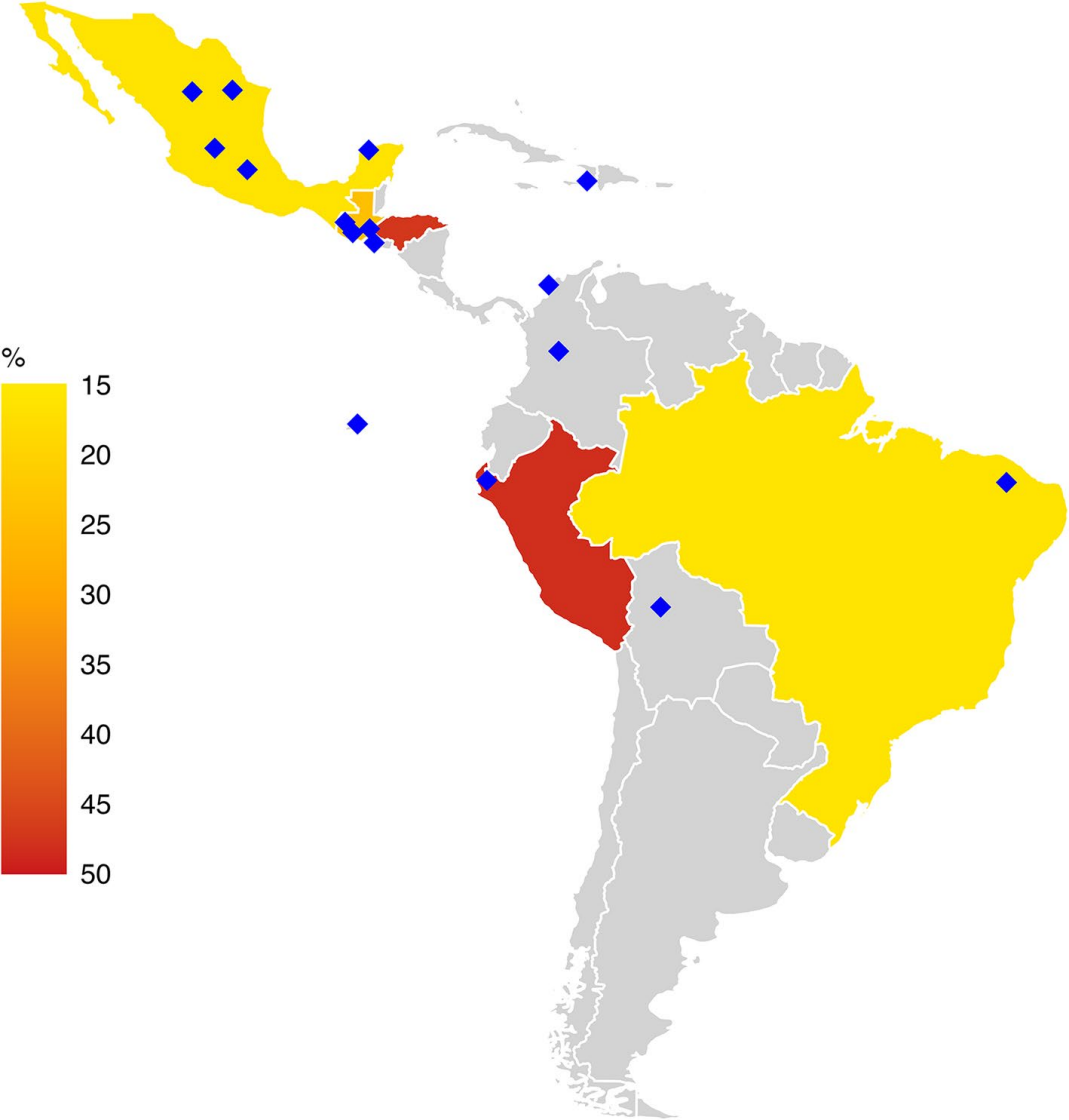
Nationally representative data have been collected using the Water Insecurity Experiences (WISE) Scales in Mexico, Honduras, Guatemala, Peru, and Brazil, indicated by shading. The WISE Scales have also been used in many site-specific studies, indicated by blue diamonds. National prevalence estimates and data sources: Mexico, 16.3%, (ENSANUT 2021); Guatemala, 24.2% (Gallup World Poll 2020), Honduras, 47.2% (Gallup World Poll 2020), Brazil, 16.1% (Gallup World Poll 2020), Peru, 48.2% (Gallup World Poll 2022). The portion of this figure based on Gallup World Poll 2020 data is replicated, with permission, from [13]

In the LAC region, household [11, 14] and individual [12, 15] versions of the WISE Scales have been applied in numerous countries, for purposes that range from research projects and development initiatives, to tracking prevalence across time (Fig. 2). Nationally representative surveys have been conducted in Mexico [16, 17], as well as in Guatemala, Honduras, Peru, and Brazil [13]; they provide the first estimates of the prevalence of experiences with water insecurity in these countries (Fig. 2). The inclusion of the HWISE Scale in Mexico’s 2021 and 2022 National Health and Nutrition Survey [16, 18] promises to yield a particularly rich set of insights into the role of water insecurity and human health and well-being. Other large studies in LAC that have used the WISE Scales, include an 11-city assessment in Colombia, and a nationally representative survey of 12,745 households carried out by the Brazilian Research Network on Food Sovereignty and Security (Rede Penssan) [19]. Site-specific studies have also been conducted throughout LAC using the WISE Scales (e.g., [20–24]).

The meeting in Mexico City offered an excellent opportunity to build and strengthen collaborations between participants and institutions; and provided a myriad of opportunities for partnering on initiatives related to water and food security, health, and policy development were identified. In the course of the seven work sessions, which took place across two days, a range of topics were discussed. These included drawing parallels to the history of the development, validation, and application of tools that measure food insecurity experiences, such as the United Nations’ Food Insecurity Experiences Scale [7]. During the “Voices of Latin America” session, researchers shared insights into inequalities in water access and use in Colombia, Mexico, and Brazil as a function of gender, ethnicity, and region. These differences had not been previously demonstrated quantitatively. In this same session it was shown that in Mexico the prevalence of water insecurity was twice as high among households with low socioeconomic status as it was among those with high socioeconomic status [17]. In a following session, further recommendations for the implementation of the scale were discussed, including its regional harmonization and a unified manual in Spanish, the broad incorporation of the WISE Scales into national surveys, and its use for program evaluation. We also discussed next avenues for scale refinement, including the development of additional levels of severity and additional items about quality and affordability.

Subsequently, participants had the opportunity to discuss the potential role of WISE data in issues such as poverty reduction, public policy development, improving health and nutrition, monitoring and evaluation, and protecting the human right to water in small groups. Throughout, there was consistency in agreement about the importance of the event and the need to implement valid and reliable tools for a more comprehensive evaluation of unmet water needs and the progress towards the human right to water.

The final session allowed the organizers to present the most important conclusions from previous sessions, and to propose next steps for collaborative WISE research and policy agenda. It was at this session that the organizers presented a public declaration that incorporated key elements recognized by consensus during the work sessions. After its reading, the statement was unanimously approved by attendees, and was later ratified in writing by the authors of this commentary. The declaration is presented below.

## Water security declaration

### Mexico city, April 2023

We, the participants in the Pan-American meeting on “The value of data on food and water insecurity experiences to improve science and policies in Latin America and the Caribbean”, held in Mexico City on April 20–21, 2023, recognize with great concern the magnitude and severity that the current water crisis keeps gaining worldwide. Despite the recognition of water security as fundamental both for life in general and specifically for food security, health, and well-being, broad sectors of the population are suffering from water insecurity, i.e., problems with reliable access to sufficient water of acceptable quality for basic domestic needs. This suffering is occurring even when indicators of physical availability and infrastructure suggest water security.

We understand the access to water as a human right (United Nations Resolution A/RES/64/292). It is therefore unacceptable that so many individuals throughout Latin America and the Caribbean lack water of acceptable quality for consumption (drinking and cooking) as well to ensure basic personal hygiene, sanitation, and to lead a productive life. This situation adds to the already worrisome inequities that characterize the reality of various peoples and nations. We recognize too that the gap in the understanding of the risks and negative impacts on human well- being caused by insecurity in access to water has been long-standing. In addition to this, we observe that the measurement tools traditionally used for the evaluation of this phenomenon ignore the experiences that a significant proportion of the population faces on a daily basis.

In this meeting we have informed ourselves about and have discussed the recent application of measurement tools focused on determining the existence of experiences related to the lack of access to water at the household and individual levels. This practice allows for the incorporation of new and highly valuable information that is closely linked to people's daily lives and the growing challenges they face when they cannot satisfy such a basic need and human right. In recent years, the use of the WISE scales in LAC countries has allowed us to assess more accurately the magnitude of this problem, and its close relationship with poverty and other inequities, including food insecurity. For this reason, we believe that this new source of information can play an important role in strengthening governance through public policies and programs that respond to a more comprehensive assessment of water insecurity.

Therefore, with emphasis on the international recognition of the human right to water, the undersigned endorse the promotion of the widespread use of the WISE scales to understand the prevalence of water insecurity, guide decisions about investments, and measure the impacts of interventions and natural shocks. We enthusiastically join and promote scientific and public policy initiatives that allow for better understanding of experiences with water access and use, in order to make progress towards Sustainable Development Goal 6, “Clean Water and Sanitation”. Given that the strengthening of public policy around water security is likely to have positive multi-sectoral impacts, e.g., from health to education and gender equity, we express our interest and willingness to deepen our collaboration with entities and initiatives aimed at promoting the use of valid and reliable measurements that support sustainable progress towards the full realization of the human right to water.

### Supplementary Information

**Additional file 1.**

**Additional file 2.**

## Data Availability

Not applicable.
