# Supplementary material for: A declaration on the value of experiential measures of food and water insecurity to improve science and policies in Latin America and the Caribbean
Source: Int J Equity Health. 2023 Sep 5;22:184. doi: 10.1186/s12939-023-01956-w (PMC10481585; doi:10.1186/s12939-023-01956-w)
Supplement: Supplementary file 1 — Additional file 1. [file 12939_2023_1956_MOESM1_ESM.docx]

**Supplementary Material**

# Declaración sobre Seguridad del Agua Ciudad de México, abril 2023

Las y los participantes en la reunión panamericana para el “*uso de datos sobre la experiencia de inseguridad alimentaria y del agua para mejorar la ciencia y las políticas en América Latina y el Caribe”*, llevado a cabo los días 20 y 21 de abril de 2023 en la Ciudad de México, con gran preocupación reconocemos la magnitud y severidad que la actual crisis del agua ha estado cobrando a nivel mundial. A pesar del reconocimiento de la seguridad del agua como factor fundamental para la vida en general y específicamente para la seguridad alimentaria, la salud y el bienestar social, amplios sectores de la población sufren de inseguridad hídrica, es decir, sufren problemas de acceso confiable a suficiente agua de calidad aceptable para las necesidades domésticas básicas. Este sufrimiento ocurre incluso cuando los indicadores de disponibilidad física e infraestructura sugieren seguridad hídrica.

Nosotros concebimos el acceso al agua como un derecho humano (Resolución de Naciones Unidas A/RES/64/292). Por ello es inaceptable que tantas personas en Latinoamérica y el Caribe no cuentan con agua de calidad adecuada para el consumo humano (beber y cocinar) y para llevar a cabo actividades básicas de higiene personal, limpieza del hogar que conducen a una vida productiva plena. Esta situación se suma a las ya de por si indignas inequidades que caracterizan la realidad de varios pueblos y naciones. Constatamos que históricamente ha existido una brecha en la comprensión de los riesgos e impactos negativos en el bienestar humano que acarrea el creciente fenómeno de la inseguridad en el acceso al agua. Aunado a ello, observamos que las herramientas de medición tradicionalmente usadas para la evaluación de este fenómeno no permiten reconocer las experiencias que cotidianamente enfrenta una importante proporción de la población.

En esta reunión nos hemos informado y hemos discutido sobre la reciente aplicación de escalas enfocadas a determinar la existencia de experiencias relacionadas con la falta de acceso al agua en el hogar y a nivel individual. Esta práctica está permitiendo incorporar novedosa y muy valiosa información, estrechamente ligada a la vida diaria de las personas y al creciente desafío que ellas enfrentan al no poder satisfacer una necesidad básica y un derecho humano. En los últimos años el uso de esas herramientas en países de América Latina y el Caribe, las escalas WISE –conocidas como escalas de experiencias en inseguridad del agua– nos ha permitido evaluar con mayor exactitud la magnitud de esa problemática y su estrecha vinculación con la pobreza, la inequidades y la inseguridad alimentaria. Por ello, consideramos que esta nueva fuente de información puede jugar un importante papel en el fortalecimiento de la gobernanza a través de políticas públicas y programas que respondan a una evaluación más integral de la inseguridad del agua.

Por lo anterior, con énfasis en el reconocimiento internacional del derecho humano al agua, las y los abajo firmantes avalamos la promoción del uso de las escalas WISE para entender la prevalencia de inseguridad de agua, guiar en la toma de decisiones sobre inversión, y medir los impactos de las intervenciones y choques naturales. Con entusiasmo nos sumamos e impulsamos

iniciativas científicas y de política pública que permitan un mejor conocimiento de las experiencias de acceso y uso de agua, en favor de una mejoría en el progreso al Objetivo de Desarrollo Sustentable 6, “Agua y Saneamiento”. Dado que el fortalecimiento de la política pública en torno a la seguridad del agua esta llamado a tener un impacto positivo en múltiples ámbitos de la sociedad, esto es, desde la salud y la educación hasta la equidad de género, expresamos nuestro interés y voluntad en profundizar nuestra colaboración con entidades e iniciativas dirigidas a impulsar el uso de mediciones válidas y confiables que apoyen el progreso sostenible hacia la consecución plena del derecho humano al agua.
