# Supplementary material for: A declaration on the value of experiential measures of food and water insecurity to improve science and policies in Latin America and the Caribbean
Source: Int J Equity Health. 2023 Sep 5;22:184. doi: 10.1186/s12939-023-01956-w (PMC10481585; doi:10.1186/s12939-023-01956-w)
Supplement: Supplementary file 2 — Additional file 2. [file 12939_2023_1956_MOESM2_ESM.docx]

**Supplementary Material**

# Declaração sobre Segurança da Água Cidade do México, abril de 2023

Os participantes da reunião pan-americana para o “*uso de dados sobre a experiência de insegurança alimentar e hídrica para melhorar a ciência e as políticas na América Latina e no Caribe*”, realizada em 20 e 21 de abril de 2023 na Cidade do México, reconhecemos com grande preocupação a magnitude e severidade que a atual crise hídrica vem ganhando em todo o mundo. Apesar do reconhecimento da segurança hídrica como fator fundamental para a vida em geral e especificamente para a segurança alimentar, saúde e bem-estar social, amplos setores da população sofrem insegurança hídrica, ou seja, problemas com acesso confiável a água suficiente e de qualidade aceitável para as necessidades domésticas básicas. Esse sofrimento ocorre mesmo quando os indicadores de disponibilidade física e infraestrutura sugerem segurança hídrica.

Nós entendemos o acesso à água como um direito humano (Resolucao das Nacoes Unidas A/RES/64/292). Isso contrasta fortemente com as evidências crescentes de que, em toda a América Latina e no Caribe, muitos indivíduos carecem de água de qualidade aceitável para consumo (beber e cozinhar), bem como para realizar higiene pessoal básica, saneamento e levar uma vida produtiva. Esta situação se soma às já indignas desigualdades que caracterizam a realidade de muitos povos e muitas nações. Verificamos que historicamente houve uma lacuna na compreensão dos riscos e impactos negativos ao bem-estar humano causados pelo crescente fenômeno da insegurança no acesso à água. Além disso, observamos que os instrumentos de medição tradicionalmente utilizados para a avaliação desse fenômeno não permitem reconhecer as experiências que uma proporção significativa da população enfrenta no dia-a-dia.

Nesta reunião nos informamos e discutimos a aplicação recente de escalas voltadas para determinar a existência de experiências relacionadas à falta de acesso à água, tanto ao nível das residências como ao nível dos indivíduos. Esta prática está a permitir a incorporação de informação nova e de grande valor, intimamente ligada ao quotidiano das pessoas e ao crescente desafio que enfrentam quando não conseguem satisfazer uma necessidade tão básica. Nos últimos anos, o uso das escalas WISE em países da America Latina e o Caribe, –conhecidas como escalas de experiências em insegurança hídrica– permitiu avaliar com mais precisão a magnitude desse problema e sua estreita relação com a pobreza, a desigualdade e a insegurança alimentar. Por isso, acreditamos que essa nova fonte de informação pode desempenhar um papel importante no fortalecimento da governança por meio de políticas e programas públicos que respondam a uma avaliação mais abrangente da insegurança hídrica.

Portanto, com ênfase no reconhecimento internacional do direito humano à água, as signatárias e os signatários abaixo, endossamos a promoção do uso das escalas WISE para entender a

prevalência do problema, orientar decisões sobre investimentos e medir os impactos de intervenções e choques naturais. Aderimos e promovemos com entusiasmo iniciativas científicas e de políticas públicas que permitam um melhor conhecimento deste fenómeno, uma melhor compreensão das experiências de acesso e uso da água, a fim de avançar em direção ao Objetivo de Desenvolvimento Sustentável 6, “Água Potável e Saneamento”. Atendendo a que o reforço da política pública em torno da segurança hídrica é chamado a ter um impacto multissetorial positivo, desde a saúde até a educação e a equidade de gênero, manifestamos o nosso interesse e vontade de aprofundar a nossa colaboração com entidades e iniciativas que visem promover a utilização de medições válidas e confiáveis, que apoiem progresso sustentável rumo à plena realização do direito humano à água.
